# Uterine cervix metastasis from primary colon adenocarcinoma: a case report and review of the literature

**DOI:** 10.1186/s13256-021-03055-2

**Published:** 2021-10-02

**Authors:** I. Sassi, M. Ghalleb, M. Chemlali, M. Mbarek, L. Charfi, R. Chargui, K. Rahal

**Affiliations:** 1 Surgical Oncology Department, Institute Salah Azaiez de Cancer, Tunis, Tunisia; 2 Pathology Department, Institute Salah Azaiez de Cancer, Tunis, Tunisia; 3 Faculté de Medicine Tunis El Manar, Tunis, Tunisia

**Keywords:** Cervix, Colorectal, Carcinoma, Metastasis

## Abstract

**Introduction:**

Metastases to the female genital tract from extragenital primary tumors are unusual. We report a rare case of uterine cervix metastasis from colon adenocarcinoma and discuss diagnostic and therapeutic issues.

**Case report:**

We report a case of a 38-year-old North African Caucasian woman treated for a non-metastatic colon adenocarcinoma. She had a sigmoidectomy and incomplete adjuvant chemotherapy. Six months later, she consulted with vaginal bleeding caused by a cervical tumor, which was confirmed to be metastatic disease, and the patient underwent decompressive and hemostatic radiotherapy.

**Conclusion:**

Uterine cervix metastasis from primary colon adenocarcinoma is rare. The resection remains the standard protocol for the local treatment of resectable metastatic disease. Otherwise, systemic therapy is the preferable option.

## Introduction

Colorectal cancer (CRC) is the third leading cause of cancer death in the world.

Approximately 50 to 60% of patients develop colorectal metastases [1, 2]. Most of these metastases develop metachronously after treatment for locoregional CRC, with the liver being the most common site of involvement [3]. The cervix is an infrequent site of metastasis. Due to the scarcity of the reports there is no real consensus for the management of this uncommon presentation. Usually they are treated with extrapolation of the data available from other more common sites of metastasis.

We Report a case a colon adenocarcinoma metastatic to the uterine cervix.

We aim through this case to report a rare case presentation and put more light into it with a review of the literature.

## Case report

A 38-year-old North African Caucasian woman with no family history of cancer presented to another teaching hospital in January 2020 with a 2-month history of abdominal pain. The physical examination revealed a distended abdomen without other abnormalities.

The patient underwent a sigmoidectomy for an obstructive tumor. The intestinal continuity was performed with an end to end colorectal mechanical anastomosis.

The histopathological analysis showed stage IIIB adenocarcinoma with a mucinous component. CT scan did not reveal systemic metastasis. Adjuvant treatment was indicated, but she received incomplete chemotherapy because of the COVID situation.

Six months later, she consulted our department for vaginal discharges and vaginal bleeding. Clinical examination showed a 5 cm cervical tumor without vaginal or parametrial involvement.

**Fig. 1:**
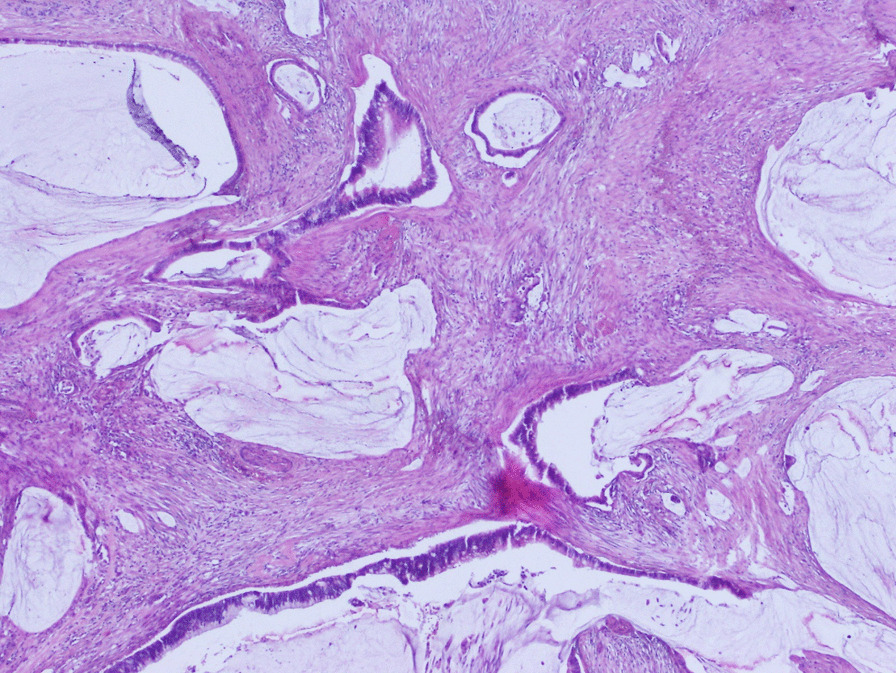
Histological slide showing the tumor with hematoxylin and eosine coloration

**Fig. 2:**
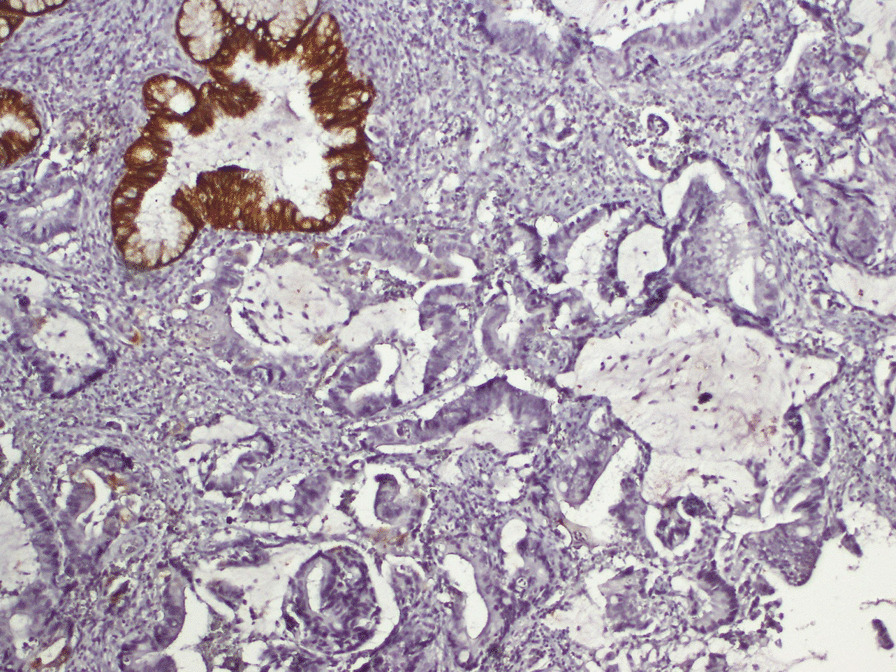
Histological slide showing CK7 negativity

**Fig. 3:**
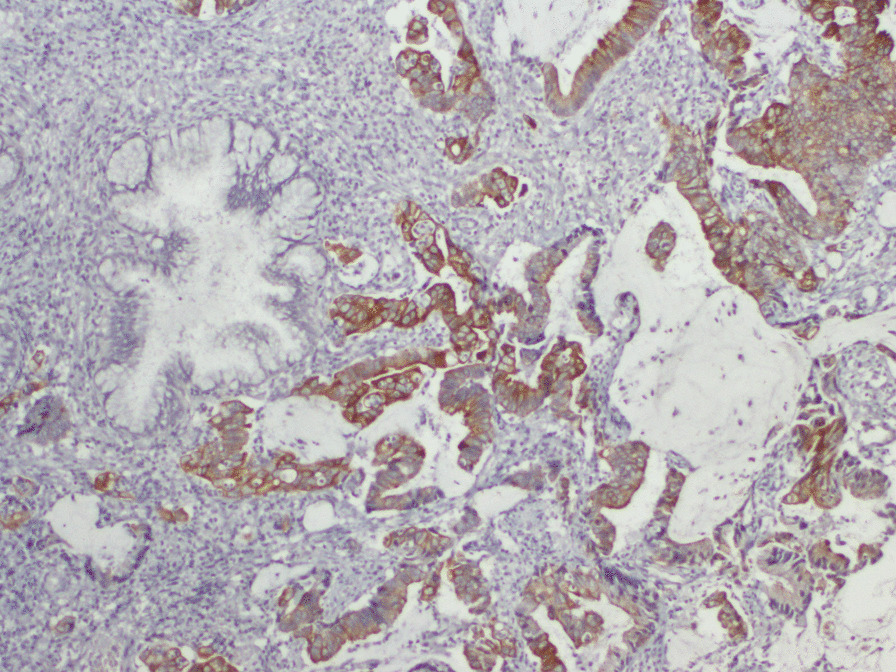
Histological slide showing CK20 positivity

The examination was otherwise regular. A cervical biopsy was suggestive of a metastatic adenocarcinoma with mucin secreting features. Immunohistochemistry showed positivity of tumor for CK20 and negative for CK7, confirming metastasis from colorectal primary (Figs.  1, 2, 3). CT scan did not show any secondary lesions, and the pelvic MRI showed a 9 × 6 × 5 cm lesion of the cervix without extension to the vagina, 3 to 4 cm pelvic lymph nodes, and a left ureteral compression with left hydronephrosis. The patient had a Double J stent placement. After discussion in a multidisciplinary meeting and the abundant vaginal bleeding, we decided to start with radiation. The patient had a protocol of 45 Gy with a good response on the cervix. The clinical examination showed the complete disappearance of the cervical tumor. The patient was scheduled for a Pelvic MRI to evaluate the locoregional response, but the patient was lost to follow up.

## Discussion

Development of metastasis to the feminine genital tract is rare, making diagnosis difficult for clinicians and pathologists. Only 3,4% of all metastases occurring in the female reproductive tract affect the cervix [4].

Metastatic carcinoma to the cervix through hematogenous or lymphatic spread is sporadic [4, 5]. Contrary to ovaries, which provide a right environment for metastatic cells, the uterine cervix is rarely the metastases site; this is due to its fibrous tissue content, small size, relatively limited blood flow, and the lymphatic vessels of the pelvis all draining away from the cervix [6].

Pérez-Montiel et al. searched for the reported cases of metastatic cancer cases of the uterine cervix registered in PubMed from 1970 until November 2009. This research concluded that the most frequent primary neoplasms derive from the ovary in 42.1% of reports, the gastrointestinal tract in 19.8%, uterine tube in 6.1%, breast in 4.5%, and primary peritoneum in 3.0% [7].

The most extensive review of cervical metastases from colorectal carcinomas to date was conducted by Nakagami et al. It showed that the interval between primary carcinoma and diagnosis of the metastasis was 17 months with a range from 0 to 60 months [8].

Pérez-Montiel et al. found that metastatic cervical disease was considered metachronous in the majority of the cases of the groups with gastric cancer (48.8%), breast (82.8%), and colorectal (78.9%), while in the group with ovarian cancer, the majority was considered synchronous (68.6%) [9].

Most cases with metastasis to the uterine cervix manifest with abnormal vaginal bleeding (62–75%) [7, 10], corroborating the findings of Pérez-Montiel et al., which found that transvaginal bleeding was most frequent in the group with colorectal cancer (64.8%) [9].

The anatomopathological examination is the definitive tool to determine the metastatic disease's true origin [11].

Adenocarcinoma histology type represents 0.42 to 11.7% of all cervix carcinomas [12]. However, metastatic adenocarcinoma incidence was 21.6 to 56.9% of cervical Adenocarcinoma [12, 13]. In these cases, Immuno-histo-chemistry (IHC) is indispensable to differentiate a genital from the extragenital origin of the uterine cervix tumor. IHC profile, CK20 positive and CK7 negative, reveals metastasis from colorectal cancer primary [14, 15]. CDX2 immunoexpression has proven to be useful in establishing gastrointestinal origin in metastatic tumors[16]. In our case, due to the lack of technical means, we were unable to test this marker on the tumor cells.

Isolated dissemination of the uterine cervix was more common in patients with ovarian and colorectal cancer (77.7 and 88.8%, respectively) compared with those with gastric or breast cancer (2.2 and 19.4%, respectively) [9].

To detect other secondary locations, the National Comprehensive Cancer Network panel recommends considering a preoperative PET/CT scan at baseline in selected cases if prior anatomic imaging indicates the presence of potentially surgically curable M1 disease. The purpose of this PET/CT scan is to evaluate for unrecognized metastatic disease that would preclude the possibility of surgical management [17]. Our patient could only afford a CT scan, which did not show any other metastatic sites.

Pelvic MRI with contrast should be considered to determine soft tissue and parametrial involvement like it has been recommended for all cervical tumors.

The latest guidelines for colon cancer patients from the National Comprehensive Cancer Network recommend resection as the standard protocol for the local treatment of resectable metastatic disease. Resection should not be undertaken unless complete removal of all known tumors is realistically possible (R0 resection) because incomplete resection or debulking (R1/R2 resection) did not show benefice [18, 19].

Otherwise, chemotherapy is increasingly weighed in specific cases to downsize colorectal metastases and convert them to a resectable status [20].

In our case, given the cervical localization and the vaginal bleeding, we opted to start with decompressive and hemostatic radiotherapy.

In all cases, a systemic therapy regimen for metastatic disease, administered for a total peri-operative treatment time of approximately 6 months, should be considered to increase the chances that residual microscopic disease will be eradicated [21, 22].

As the role of targeted therapy for the treatment of metastatic CRC has become essential, the NCCN Panel recommends the determination of tumor gene status for KRAS/RAS and BRAF mutations, as well as HER2 amplifications.

More recent favorable results of randomized clinical trials evaluating FOLFIRI, FOLFOX, or FOLFOXIRI in combination with anti-epidermal growth factor receptor (EGFR) inhibitors for converting unresectable disease to resectable disease have been reported [23].

## Conclusion

Cervical, colorectal cancer metastasis is rare and often hard to diagnose. These metastases should be investigated when patients with a history of primary gastrointestinal tract carcinoma present abnormal vaginal bleeding or discharge. For an accurate diagnosis of the origin of uterine cervical adenocarcinoma cancer, immunohistochemistry should be performed. For adequate therapeutic management, it is essential to identify other metastatic sites. The resection remains the standard protocol for the local treatment of resectable metastatic disease. Otherwise, systemic therapy is the preferable option.

## Data Availability

All the data used was taken from the patient's medical folder available at our institution's archive.
